# Supplementary material for: Long-term clinical outcomes after deployment of femoral vascular closure devices in coronary angiography and percutaneous coronary intervention: an observational single-centre registry follow-up
Source: BMJ Open. 2014 Jun 12;4(6):e005126. doi: 10.1136/bmjopen-2014-005126 (PMC4067826; doi:10.1136/bmjopen-2014-005126)
Supplement: Author's manuscript [file bmjopen-2014-005126.draft_revisions.pdf]

Long Term Clinical Outcomes After Deployment of Femoral Vascular Closure Devices in Coronary Angiography and Percutaneous Coronary Intervention – A Follow Up Cohort Study

Journal:	*BMJ Open*
Manuscript ID:	bmjopen-2014-005126
Article Type:	Research
Date Submitted by the Author:	25-Feb-2014

Complete List of Authors:	Lee, Wai Luen Stephen; Queen Mary Hospital, Medicine<br>TAM, Frankie; Queen Mary Hospital, Univeristy of Hong Kong, Medicine<br>WONG, Michael; Queen Mary Hospital, Univeristy of Hong Kong, Medicine<br>KONG, Shun-Ling; Queen Mary Hospital, Univeristy of Hong Kong, Medicine<br>YUNG, Arthur; Queen Mary Hospital, Univeristy of Hong Kong, Medicine<br>WONG, Anthony; Queen Mary Hospital, University of Hong Kong, Medicine<br>Chiu, SY; Queen Mary Hospital, Univeristy of Hong Kong, Medicine<br>LAM, Simon; Queen Mary Hospital, Univeristy of Hong Kong, Medicine<br>CHAN, Kelvin; Queen Mary Hospital, Univeristy of Hong Kong, Medicine<br>CHAN, Raymond; Queen Mary Hospital, University of Hong Kong, Medicine

**Primary Subject Heading**:	Cardiovascular medicine
Secondary Subject Heading:	Cardiovascular medicine
Keywords:	Coronary intervention < CARDIOLOGY, Coronary heart disease < CARDIOLOGY, Adult cardiology < CARDIOLOGY

For peer review only

SCHOLARONE™
Manuscripts

Long Term Clinical Outcomes After Deployment of Femoral Vascular Closure Devices in Coronary Angiography and Percutaneous Coronary Intervention – A Follow Up Cohort Study

Stephen Wai-Luen Lee, Chor-Cheung Tam, Ka-Lam Wong, Shun-Ling Kong, See-Yue Yung, Yiu-Tung Wong, Suk-Yee Chiu, Cheung-Chi Lam, Ki-Wan Chan, Hon-Wah Chan
Queen Mary Hospital, University of Hong Kong

Stephen Wai-Luen Lee
Division of Cardiology, Queen Mary Hospital, University of Hong Kong

Chor-Cheung Tam
Division of Cardiology, Queen Mary Hospital, University of Hong Kong

Ka-Lam Wong
Division of Cardiology, Queen Mary Hospital, University of Hong Kong

Shun-Ling Kong
Division of Cardiology, Queen Mary Hospital, University of Hong Kong

See-Yue Yung
Division of Cardiology, Queen Mary Hospital, University of Hong Kong

Yiu-Tung Wong
Division of Cardiology, Queen Mary Hospital, University of Hong Kong

Suk-Yee Chiu
Division of Cardiology, Queen Mary Hospital, University of Hong Kong

Cheung-Chi Lam
Division of Cardiology, Queen Mary Hospital, University of Hong Kong

Ki-Wan Chan
Division of Cardiology, Queen Mary Hospital, University of Hong Kong

Hon-Wah Chan
Division of Cardiology, Queen Mary Hospital, University of Hong Kong

For peer review only

For peer review only - http://bmjopen.bmj.com/site/about/guidelines.xhtml

Correspondence:
Stephen Wai-Luen Lee
1/F, A block, Queen Mary Hospital, 102, Pok Fu Lam Road, Hong Kong,
E-mail: leewls@ha.org.hk
Fax: 852-22555411

Keywords
Coronary intervention, coronary artery disease, adult cardiology, vascular closure device

Word count
1703

For peer review only

For peer review only - http://bmjopen.bmj.com/site/about/guidelines.xhtml

ABSTRACT:

Objectives: There are few data evaluating the long term effect of femoral vascular closure devices (FCD) on patients' clinical outcome. We aim to evaluate the incidence of peripheral vascular disease (PVD) in patients received FCD following its deployment in coronary angiography and percutaneous coronary intervention (PCI) procedures.

Design and setting: Single center, prospective cohort study

Participants: From June 2000 to September 2004, 265 patients who received FCD after coronary angiography and PCIs were enrolled into study.

Outcome measures: Clinical follow up (using Rutherford's categories of claudication), ankle brachial index (ABI) and Duplex ultrasound of femoral arteries (using non-accessed side as control) were performed to evaluate the presence of PVD.

Results: The mean follow up interval was 3320±628 days. 1 patient (0.4%) suffered from Grade 2 claudication and another (0.4%) suffered from Grade 1 claudication. The mean ABI of accessed side and non-accessed side were 1.06±0.13 and 1.08±0.11

For peer review only

For peer review only - http://bmjopen.bmj.com/site/about/guidelines.xhtml

respectively, ($p = 0.17$). For duplex ultrasound, mean common femoral artery peak systolic velocity of accessed side and non-accessed side were 87.4±22.3 cm/s and 87.7±22.1 cm/s respectively, ($p = 0.73$); mean superficial femoral artery peak systolic velocity of accessed side and non-accessed side were 81.4±20.1 cm/s and 81.31±17.8 cm/s respectively, ($p = 0.19$).

Conclusion: The use of femoral vascular closure device after coronary angiogram and PCI is safe and does not increase the long term risk of peripheral vascular disease.

For peer review only

ARTICLE SUMMARY

Strengths and limitations of current study

- This study showed no increase in incidence of clinically significant peripheral vascular disease after use of femoral vascular closure device in long term follow up (median follow up time approaching 10 years).
- The use of duplex ultrasound showed no evidence of adverse effect of femoral vascular closure device on accessed femoral artery
- The lack of baseline ultrasound before vascular closure device precluded precise evaluation of effect of vascular remodeling of femoral artery after collagen plug and suture deployment.
- Current study results may not be able to generalize to other vascular closure device or in setting of large size femoral access as in structural heart disease intervention.

For peer review only

For peer review only - http://bmjopen.bmj.com/site/about/guidelines.xhtml

Introduction

Despite the increasing trend of utilizing radial access for coronary angiogram and percutaneous coronary intervention (PCI), femoral artery wound hemostasis is still an integral part in the field of interventional cardiology. Femoral vascular closure devices (FCD) are designed specifically to facilitate arteriotomy closure which has been shown to allow shorter hemostasis time and early ambulation. However, there is no data to demonstrate the superiority of the safety of FCD compared with manual compression and long term effect of FCD on peripheral vasculature has not been extensively investigated. Our group has published data [1] on the in-hospital and 1 year clinical outcomes in patients received FCD after coronary angiogram and PCIs before and we perform this follow up study to evaluate the long term clinical data in the same group of patients.

Methods:

The design of the study has been described in elsewhere [1] . From June 2000 to September 2004, 265 patients who successfully received FCDs after coronary angiogram and PCIs were enrolled for study. The in-hospital and 1 year outcome was evaluated by clinical assessment and duplex ultrasound. For the current study, patients were continued to be followed up in our clinic while clinical symptom and

For peer review only - http://bmjopen.bmj.com/site/about/guidelines.xhtml

sign of limb ischemia were assessed by Rutherford's categories of claudication. Furthermore, ankle brachial index (ABI) was measured by using sphygmomanaometer and vascular doppler probe (Hadeco minidop ES-100VX). Patients were instructed to lie comfortably on bed and blood pressure cuff was applied to patients' arms and doppler probe was positioned at the brachial artery to obtain the systolic brachial pressure while the cuff was then placed at calfs with doppler probe positioned at dorsalis pedis or posterior tibial artery (whichever is higher) to obtain the ankle systolic pressure. In addition, duplex ultrasound using a high frequency linear array transducer was performed to assess the peak systolic velocity at common femoral artery and superficial femoral artery. Non-accessed side was used as control to compare values of ABI and peak systolic velocity to the side where FCDs were deployed. The study was approved by the local ethics committee.

Continuous variables were expressed as mean standard deviation. Dichotomous variables were expressed as counts and percentage. Statistical comparisons were performed using Student's t test or Wilcoxon signed rank test for continuous variables as appropriate while Fisher's exact test was used for categorical variables. A P value < 0.05 was considered statistically significant. All statistical analyses were performed using SPSS for Windows (version 19.0, SPSS Inc.-------USA).

For peer review only - http://bmjopen.bmj.com/site/about/guidelines.xhtml

Results:

265 patients entered into study, the baseline clinical and procedural characteristics were shown in table 1. The mean age of patients was 61.8±10.4 years old with 80% were male. Vascular closure devices were used in 235 PCI (88.7%) and 30 (11.3%) coronary angiographic procedures. 60 procedures (22.6%) used Angioseal whereas Perclose closure device was deployed in 205 procedures (77.4%). Most of the vascular closure devices used were 6F in size. Patients were continued to be followed up and for the current study, 233 patients were recruited for analysis while ABI and duplex ultrasound studies were performed in 145 patients (Figure 1).

Table 1 Baseline characteristics

Clinical characteristics		Procedural variables	
Mean age (years)	61.8±10.4	Coronary angiogram, n (%)	30 (11.3)
Gender-male, (%)	213 (80.4)	PCI, n (%)	235(88.7)
Current smoker, n (%)	54 (20.4)	Angioseal, n (%)	60 (22.6)
Diabetes mellitus, n (%)	63 (23.8)	Perclose, n (%)	205 (77.4)
Hypertension, n (%)	130 (49.1)	Sheath size	
Hyperlipidemia, n (%)	147 (55.5)	6F, n (%)	237 (89.4)
Peripheral vascular disease, n (%)	8 (3.0)	7F, n (%)	2 (0.8)
		8F, n (%)	26 (9.8)
Renal failure, n (%)	7 (2.6)	Mean ACT, seconds	346±61
Left ventricular function (%)	64.9±14.3	Mean procedural time for archieving hemostasis, seconds	147±121
Body mass index, kg/m2	24.9±3.1		
		Right side : left side	256 : 7

For peer review only - http://bmjopen.bmj.com/site/about/guidelines.xhtml

For peer review only

The mean follow up time was 3320±628 days. Out of 233 patients with long term follow up, there was 1 case (0.4%) of Rutherford grade 2 claudication with a 79-year-old lady with end stage renal failure complained of bilateral calf claudication. There was another case (0.4%) of bilateral Rutherford grade 1 claudication in a 78-year-old gentleman with bilateral lower limb peripheral vascular disease (PVD) and duplex ultrasound confirmed right superficial femoral artery (SFA) occlusion and left common femoral artery (CFA) 70% stenosis. The remaining patients were free of symptomatic PVD (Table 2).

Table 2 also showed the ABI and duplex ultrasound results on long term follow up. The ABI of accessed and non-accessed sites were 1.06±0.13 and 1.08±0.11 respectively. Duplex ultrasound measured peak systolic velocity of common femoral artery (CFA) in accessed and non-accessed sites were 87.43±22.34 cm/s and 87.69±22.06 cm/s respectively and of superficial femoral artery (SFA) were 81.40±20.85 cm/s and 81.31±17.80 cm/s respectively. There was no statistically significant difference in above parameters between accessed and non-accessed sites. Nevertheless, the mean end-diastolic diameter (EDD) of CFA and SFA in accessed sites was significantly larger than that in non-accessed sites (CFA: 8.12±1.27mm vs

For peer review only - http://bmjopen.bmj.com/site/about/guidelines.xhtml

7.80±1.16mm, *p*<0.01; SFA: 6.58±0.96mm vs 6.38±0.95mm, *p*<0.01).

Table 2 Follow up results

**Clinical Outcomes**			
**Rutherford's grade of claudication**			
**Grade 0, n (%)**	231 (99.2)		
**Grade 1, n (%)**	1 (0.4)		
**Grade 2, n (%)**	1 (0.4)		
	Access site	Control site	*p*
**Ankle Brachial Index (ABI)**	1.06±0.13	1.08±0.11	0.17
**Duplex ultrasound**	Access site	Control site	*p*
**CFA peak systolic velocity, cm/s**	87.43±22.34	87.69±22.06	0.73
**SFA peak systolic velocity, cm/s**	81.40±20.85	81.31±17.80	0.19
**CFA end-diastolic diameter, cm**	8.12±1.27	7.80±1.16	<0.01
**SFA end-diastolic diameter, cm**	6.58±0.96	6.38±0.95	<0.01

CFA: common femoral artery

SFA: superficial femoral artery

Data was also analysed between angioseal and perclose subgroup and results were shown in table 3. Baseline clinical and procedural characteristics were similar between both groups while patients received perclose had non-significantly higher peak systolic velocity in both CFA and SFA. The mean EDD of CFA in accessed sites was significantly larger in patients received perclose device than those received angioseal (perclose: 8.25±1.28mm vs angioseal: 7.58±1.10mm, p =0.02) but it is not the case for mean EDD of SFA.

For peer review only - http://bmjopen.bmj.com/site/about/guidelines.xhtml

Table 3 Angioseal vs Perclose

	Angioseal (n=60)	Perclose (n=203)	*P*
**Baseline characteristics**			
**Mean age (years)**	62.4±10.8	61.7±10.3	0.67
**Gender-male, (%)**	50 (83.3)	162 (79.8)	0.71
**Diabetes mellitus, n (%)**	19 (31.7)	44 (21.7)	0.12
**Hypertension, n (%)**	27 (45.0)	101 (49.8)	0.56
**Body mass index, kg/m2**	24.6±3.4	24.9±3.1	0.53
**Coronary angiogram, n (%)**	5 (8.5)	25 (12.5)	0.49
**PCI, n (%)**	55 (91.5)	178 (87.5)	0.49
**Parameters on accessed side**			
**Ankle Brachial Index (ABI)**	1.08±0.09	1.06±0.14	0.98
**CFA peak systolic velocity, cm/s**	84.66±15.09	88.07±23.70	0.55
**SFA peak systolic velocity, cm/s**	80.95±21.10	81.50±20.89	0.79
**CFA end-diastolic diameter, cm**	7.58±1.10	8.25±1.28	0.02
**SFA end-diastolic diameter, cm**	6.37±1.09	6.63±0.93	0.25

CFA: common femoral artery; SFA: superficial femoral artery

PCI: percutaneous coronary intervention

Discussion

To our knowledge, this is the first study to evaluate the long term outcome of femoral vascular closure device. In the study published earlier, we demonstrated the favorable in-hospital and 1 year outcome of usage of FCD in our patients while this study showed nil evidence of increase in risk of lower limb peripheral vascular disease after a mean follow up of almost 10 years. Most previous studies including meta-analysis [2-5] comparing FCD and manual compression did not confirm a significant advantage of FCD in reducing short term vascular complications apart

For peer review only - http://bmjopen.bmj.com/site/about/guidelines.xhtml

from the shortened hemostasis time and faster ambulation of FCD over manual compression. Theoretically, use of collagen plug and suture mediated vascular closure after femoral arterial access can impinge on the common femoral artery and potentially lead to flow disturbance which accelerates atherosclerosis. Our study using the non-accessed side as control and it identified nil increased incidence of clinical PVD by symptom, ABI and duplex ultrasound. On the contrary, the EDD of CFA and SFA in accessed sites in our patients were larger than that in non-accessed sites. Since we did not have baseline ultrasound of bilateral femoral arteries before device deployment, we cannot ascertain whether the discrepancy is due to nature difference or device-related injury and positive remodeling. It has been shown in a study that diameter of right CFA is larger than that of left CFA [6],with a mean difference of 0.26mm which is similar to the difference of 0.32mm between our patients' accessed (>99% right side) and non-accessed sites. Further studies are needed to verify whether femoral closure device will have impact on femoral artery positive remodeling.

In animal model [7], collagen plug produced more intense inflammatory reaction of the tissue surrounding the femoral arteries when compared to suture mediated device but this did not lead to higher incidence of clinical PVD and duplex ultrasound derived flow abnormalities in our patients received Angioseal. On the other hand, the

For peer review only - http://bmjopen.bmj.com/site/about/guidelines.xhtml

diameter of the accessed CFA is larger in perclose than in angioseal. We cannot determine whether a more intense inflammation with Angioseal gives rise to a smaller vessel caliber in the long run or Perclose triggers a more pronounced positive remodeling. Nonetheless, this interesting finding highlights tissue remodeling may differ between different mechanisms of vascular healing.

Limitations

We did not have baseline duplex ultrasound in our patients and we used non-accessed side as control for which we assumed the baseline appearance of both sides of femoral arteries were identical. In addition, this was a single center study and the sample size was relatively small which may be underpowered to detect rare vascular events. Furthermore, more than one third of patients did not consent for ABI and duplex ultrasound measurement, making the data less representative. Moreover, VCD used in current study were either Angioseal or Perclose from 6 to 8 French and hence results may not be able to generalize to other VCD or in setting of larger size femoral access as in structural heart disease intervention.

Conclusions

Use of femoral vascular closure devices was safe and did not increase the incidence

For peer review only - http://bmjopen.bmj.com/site/about/guidelines.xhtml

of lower limb peripheral vascular disease on long term follow up.

Contributors:

SWLL initiated the concept of work, performed the analyses in conjunction with coauthors and wrote and revised the manuscript. CCT, KLW, SLK, SYY, YTW and SYC performed clinical and ultrasound assessment. All authors contributed to the revisions of the manuscript and the interpretation of the findings.

Funding statement:

This research received no specific grant from any funding agency in the public, commercial or not-for-profit sectors

Competing interests:

None

For peer review only - http://bmjopen.bmj.com/site/about/guidelines.xhtml

References

1 Lee SW, Ho HH, Kong SL, et al. Long term clinical outcomes after deployment of femoral vascular closure devices in coronary angiography and percutaneous coronary intervention. Catheterization and cardiovascular interventions : official journal of the Society for Cardiac Angiography & Interventions. 2010;75(3):345-8. Epub 2009/11/26.

2 Chevalier B, Lancelin B, Koning R, et al. Effect of a closure device on complication rates in high-local-risk patients: results of a randomized multicenter trial. Catheterization and cardiovascular interventions : official journal of the Society for Cardiac Angiography & Interventions. 2003;58(3):285-91. Epub 2003/02/21.

3 Gerckens U, Cattelaens N, Lampe EG, et al. Management of arterial puncture site after catheterization procedures: evaluating a suture-mediated closure device. The American journal of cardiology. 1999;83(12):1658-63. Epub 1999/07/07.

4 Nikolsky E, Mehran R, Halkin A, et al. Vascular complications associated with arteriotomy closure devices in patients undergoing percutaneous coronary procedures: a meta-analysis. Journal of the American College of Cardiology. 2004;44(6):1200-9. Epub 2004/09/15.

5 Vaitkus PT. A meta-analysis of percutaneous vascular closure devices after diagnostic catheterization and percutaneous coronary intervention. The Journal of invasive cardiology. 2004;16(5):243-6. Epub 2004/05/21.

For peer review only - http://bmjopen.bmj.com/site/about/guidelines.xhtml

6 Eisen LA, Minami T, Sekiguchi H, et al. Ultrasound demonstration of asymmetry between the left and right femoral and radial arteries. Chest. 2006;130(4):201s-s.

7 Gargiulo NJ, 3rd, Veith FJ, Ohki T, et al. Histologic and duplex comparison of the perclose and angio-seal percutaneous closure devices. Vascular. 2007;15(1):24-9. Epub 2007/03/27.

For peer review only

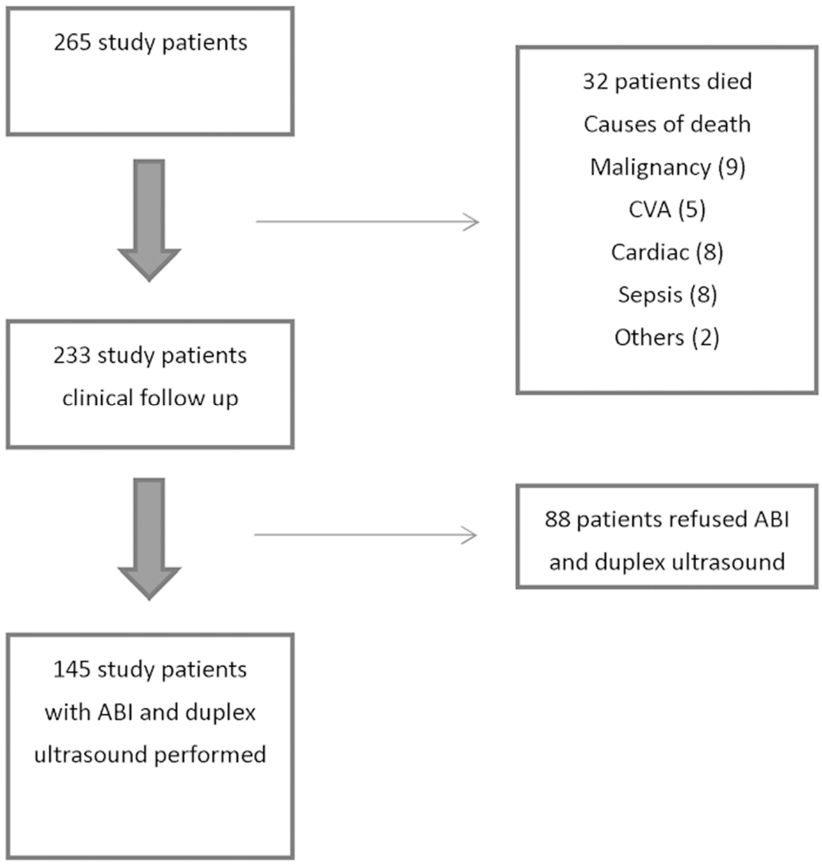

Figure 1 Study patients

70x86mm (300 x 300 DPI)

STROBE 2007 (v4) Statement—Checklist of items that should be included in reports of *cohort studies*

Section/Topic	Item #	Recommendation	Reported on page #
**Title and abstract**	1	(*a*) Indicate the study's design with a commonly used term in the title or the abstract	3, 6
		(*b*) Provide in the abstract an informative and balanced summary of what was done and what was found	3, 4
**Introduction**			
Background/rationale	2	Explain the scientific background and rationale for the investigation being reported	6
Objectives	3	State specific objectives, including any prespecified hypotheses	6
**Methods**			
Study design	4	Present key elements of study design early in the paper	6, reference 1
Setting	5	Describe the setting, locations, and relevant dates, including periods of recruitment, exposure, follow-up, and data collection	6, reference 1
Participants	6	(*a*) Give the eligibility criteria, and the sources and methods of selection of participants. Describe methods of follow-up	6, reference 1
		(*b*) For matched studies, give matching criteria and number of exposed and unexposed	
Variables	7	Clearly define all outcomes, exposures, predictors, potential confounders, and effect modifiers. Give diagnostic criteria, if applicable	6, 7
Data sources/ measurement	8*	For each variable of interest, give sources of data and details of methods of assessment (measurement). Describe comparability of assessment methods if there is more than one group	6, 7
Bias	9	Describe any efforts to address potential sources of bias	
Study size	10	Explain how the study size was arrived at	
Quantitative variables	11	Explain how quantitative variables were handled in the analyses. If applicable, describe which groupings were chosen and why	7
Statistical methods	12	(*a*) Describe all statistical methods, including those used to control for confounding	7
		(*b*) Describe any methods used to examine subgroups and interactions	
		(*c*) Explain how missing data were addressed	
		(*d*) If applicable, explain how loss to follow-up was addressed	
		(*e*) Describe any sensitivity analyses	
**Results**			
Participants	13*	(a) Report numbers of individuals at each stage of study—eg numbers potentially eligible, examined for eligibility, confirmed	Figure 1

For peer review only

		eligible, included in the study, completing follow-up, and analysed	
		(b) Give reasons for non-participation at each stage	Figure 1
		(c) Consider use of a flow diagram	Figure 1
Descriptive data	14*	(a) Give characteristics of study participants (eg demographic, clinical, social) and information on exposures and potential confounders	Table 1
		(b) Indicate number of participants with missing data for each variable of interest	
		(c) Summarise follow-up time (eg, average and total amount)	9
Outcome data	15*	Report numbers of outcome events or summary measures over time	9
Main results	16	(*a*) Give unadjusted estimates and, if applicable, confounder-adjusted estimates and their precision (eg, 95% confidence interval). Make clear which confounders were adjusted for and why they were included	
		(*b*) Report category boundaries when continuous variables were categorized	
		(*c*) If relevant, consider translating estimates of relative risk into absolute risk for a meaningful time period	
Other analyses	17	Report other analyses done—eg analyses of subgroups and interactions, and sensitivity analyses	
**Discussion**			
Key results	18	Summarise key results with reference to study objectives	11
**Limitations**			
Interpretation	20	Give a cautious overall interpretation of results considering objectives, limitations, multiplicity of analyses, results from similar studies, and other relevant evidence	11, 12, 13
Generalisability	21	Discuss the generalisability (external validity) of the study results	12
**Other information**			
Funding	22	Give the source of funding and the role of the funders for the present study and, if applicable, for the original study on which the present article is based	13

*Give information separately for cases and controls in case-control studies and, if applicable, for exposed and unexposed groups in cohort and cross-sectional studies.

Note: An Explanation and Elaboration article discusses each checklist item and gives methodological background and published examples of transparent reporting. The STROBE checklist is best used in conjunction with this article (freely available on the Web sites of PLoS Medicine at http://www.plosmedicine.org/, Annals of Internal Medicine at http://www.annals.org/, and Epidemiology at http://www.epidem.com/). Information on the STROBE Initiative is available at www.strobe-statement.org.

For peer review only

Long Term Clinical Outcomes After Deployment of Femoral Vascular Closure Devices in Coronary Angiography and Percutaneous Coronary Intervention – An observational single center registry follow up

Journal:	*BMJ Open*
Manuscript ID:	bmjopen-2014-005126.R1
Article Type:	Research
Date Submitted by the Author:	02-May-2014
Complete List of Authors:	Lee, Wai Luen Stephen; Queen Mary Hospital, Medicine<br>TAM, Frankie; Queen Mary Hospital, Univeristy of Hong Kong, Medicine<br>WONG, Michael; Queen Mary Hospital, Univeristy of Hong Kong, Medicine<br>KONG, Shun-Ling; Queen Mary Hospital, Univeristy of Hong Kong, Medicine<br>YUNG, Arthur; Queen Mary Hospital, Univeristy of Hong Kong, Medicine<br>WONG, Anthony; Queen Mary Hospital, University of Hong Kong, Medicine<br>Chiu, SY; Queen Mary Hospital, Univeristy of Hong Kong, Medicine<br>LAM, Simon; Queen Mary Hospital, Univeristy of Hong Kong, Medicine<br>CHAN, Kelvin; Queen Mary Hospital, Univeristy of Hong Kong, Medicine<br>CHAN, Raymond; Queen Mary Hospital, University of Hong Kong, Medicine
**Primary Subject Heading**:	Cardiovascular medicine
Secondary Subject Heading:	Cardiovascular medicine
Keywords:	Coronary intervention < CARDIOLOGY, Coronary heart disease < CARDIOLOGY, Adult cardiology < CARDIOLOGY

For peer review only

Long Term Clinical Outcomes After Deployment of Femoral Vascular Closure Devices in Coronary Angiography and Percutaneous Coronary Intervention – An observational single center registry follow up

Stephen Wai-Luen Lee, Chor-Cheung Tam, Ka-Lam Wong, Shun-Ling Kong, See-Yue Yung, Yiu-Tung Wong, Suk-Yee Chiu, Cheung-Chi Lam, Ki-Wan Chan, Hon-Wah Chan
Queen Mary Hospital, University of Hong Kong

Stephen Wai-Luen Lee
Division of Cardiology, Queen Mary Hospital, University of Hong Kong

Chor-Cheung Tam
Division of Cardiology, Queen Mary Hospital, University of Hong Kong

Ka-Lam Wong
Division of Cardiology, Queen Mary Hospital, University of Hong Kong

Shun-Ling Kong
Division of Cardiology, Queen Mary Hospital, University of Hong Kong

See-Yue Yung
Division of Cardiology, Queen Mary Hospital, University of Hong Kong

Yiu-Tung Wong
Division of Cardiology, Queen Mary Hospital, University of Hong Kong

Suk-Yee Chiu
Division of Cardiology, Queen Mary Hospital, University of Hong Kong

Cheung-Chi Lam
Division of Cardiology, Queen Mary Hospital, University of Hong Kong

Ki-Wan Chan
Division of Cardiology, Queen Mary Hospital, University of Hong Kong

Hon-Wah Chan
Division of Cardiology, Queen Mary Hospital, University of Hong Kong

For peer review only

For peer review only - http://bmjopen.bmj.com/site/about/guidelines.xhtml

Correspondence:
Stephen Wai-Luen Lee
1/F, A block, Queen Mary Hospital, 102, Pok Fu Lam Road, Hong Kong,
E-mail: leewls@ha.org.hk
Fax: 852-22555411

Keywords
Coronary intervention, coronary artery disease, adult cardiology, vascular closure device

Word count
1851

For peer review only

For peer review only - http://bmjopen.bmj.com/site/about/guidelines.xhtml

ABSTRACT:

Objectives: There are few data evaluating the long term effect of femoral vascular closure devices (FCD) on patients' clinical outcome. We aim to evaluate the incidence of peripheral vascular disease (PVD) in patients received FCD following its deployment in coronary angiography and percutaneous coronary intervention (PCI) procedures.

Design and setting: Observational study of a single center registry

Participants: From June 2000 to September 2004, 265 patients who received FCD after coronary angiography and PCIs were enrolled into study.

Outcome measures: Clinical follow up (using Rutherford's categories of claudication), ankle brachial index (ABI) and Duplex ultrasound of femoral arteries (using non-accessed side as control) were performed to evaluate the presence of PVD.

Results: The mean follow up interval was 3320±628 days. 1 patient (0.4%) suffered from Grade 2 claudication and another (0.4%) suffered from Grade 1 claudication. The mean ABI of accessed side and non-accessed side were 1.06±0.13 and 1.08±0.11

For peer review only - http://bmjopen.bmj.com/site/about/guidelines.xhtml

respectively, (p = 0.17). For duplex ultrasound, mean common femoral artery peak systolic velocity of accessed side and non-accessed side were 87.4±22.3 cm/s and 87.7±22.1 cm/s respectively, (p = 0.73); mean superficial femoral artery peak systolic velocity of accessed side and non-accessed side were 81.4±20.1 cm/s and 81.31±17.8 cm/s respectively, (p = 0.19).

For peer review only

Conclusion: The use of femoral vascular closure device after coronary angiogram and PCI is safe and does not increase the long term risk of peripheral vascular disease.

For peer review only - http://bmjopen.bmj.com/site/about/guidelines.xhtml

ARTICLE SUMMARY

Strengths and limitations of current study

- This study showed no increase in incidence of clinically significant peripheral vascular disease after use of femoral vascular closure device in long term follow up (median follow up time approaching 10 years).
- The use of duplex ultrasound showed no evidence of adverse effect of femoral vascular closure device on accessed femoral artery
- The lack of baseline ultrasound before vascular closure device precluded precise evaluation of effect of vascular remodeling of femoral artery after collagen plug and suture deployment.
- Current study results may not be able to generalize to other vascular closure device or in setting of large size femoral access as in structural heart disease intervention.

For peer review only

For peer review only - http://bmjopen.bmj.com/site/about/guidelines.xhtml

Introduction

Despite the increasing trend of utilizing radial access for coronary angiogram and percutaneous coronary intervention (PCI), femoral artery access hemostasis is still an integral part in the field of interventional cardiology. Femoral vascular closure devices (FCD) are designed specifically to facilitate arteriotomy closure which has been shown to allow shorter hemostasis time and early ambulation [1-3]. However, there is no data to demonstrate the superiority of FCD in reducing vascular complications when compared with manual compression [1, 4, 5] and long term effect of FCD on peripheral vasculature has not been extensively investigated, apart from scanty case report describing the occurrence of delayed claudication post FCD use [6, 7]. Particularly, the impingement of femoral artery by FCD with subsequent inflammation and remodeling can take place for years which potentially cause vascular stenosis. Our group has previously published data [8] on the in-hospital and 1 year clinical outcomes in patients received FCD after coronary angiogram and PCIs before and we performed this follow up study to evaluate the incidence of lower limb peripheral vascular disease (PVD) in the same group of patients in long run.

Methods:

The design of the study has been described in elsewhere [8]. In brief, from June 2000

For peer review only - http://bmjopen.bmj.com/site/about/guidelines.xhtml

to September 2004, 265 patients who successfully received FCDs after coronary angiogram and PCIs were enrolled for study. Femoral arteriogram were routinely performed before consideration of arterial closure and two types of FCD namely Angioseal (St. Jude Medical, Minnesota, MN) and Perclose (Abbott, Abbott Park, IL) closure devices were used. Angioseal is a collagen-mediated device which utilizes a bioabsorbable collagen sponge to seal the arteriotomy site while Perclose achieves hemostasis by delivering a knot to suture the arteriotomy.

For the long term registry follow up, all patients were seen in our clinic every four to six months and occurrence of any vascular complications was recorded. Clinical symptom and sign of lower limb ischemia were assessed by Rutherford's grade of claudication [9] and ankle brachial index (ABI) was measured by using sphygmomanaometer and vascular doppler probe (Hadeco minidop ES-100VX). Patients were instructed to lie comfortably on bed and blood pressure cuff was applied to patients' arms and doppler probe was positioned at the brachial artery to obtain the systolic brachial pressure while the cuff was then placed at calfs with doppler probe positioned at dorsalis pedis or posterior tibial artery (whichever is higher) to obtain the ankle systolic pressure. In addition, duplex ultrasound using a high frequency linear array transducer was performed to assess the peak systolic velocity at common femoral artery and superficial femoral artery. Non-accessed side

For peer review only - http://bmjopen.bmj.com/site/about/guidelines.xhtml

was used as control to compare values of ABI and peak systolic velocity to the side where FCDs were deployed. The study was approved by the local ethics committee.

Continuous variables were expressed as the mean and standard deviation. Dichotomous variables were expressed as counts and percentage. Statistical comparisons were performed using Student's t test or Wilcoxon signed rank test for continuous variables as appropriate while Fisher's exact test was used for categorical variables. A P value < 0.05 was considered statistically significant. All statistical analyses were performed using SPSS for Windows (version 19.0, SPSS Inc.-------USA).

Results:

265 patients entered into study, the baseline clinical and procedural characteristics were shown in table 1. The mean age of patients was 61.8±10.4 years old with 80% were male. Vascular closure devices were used in 235 PCI (88.7%) and 30 (11.3%) coronary angiographic procedures. 60 procedures (22.6%) used Angioseal whereas Perclose closure device was deployed in 205 procedures (77.4%). Most of the vascular closure devices used were 6F in size. Patients were continued to be followed up and for the current study, 233 patients were recruited for analysis while ABI and duplex ultrasound studies were performed in 145 patients (Figure 1).

For peer review only - http://bmjopen.bmj.com/site/about/guidelines.xhtml

Table 1 Baseline characteristics

Clinical characteristics		Procedural variables	
Mean age (years)	61.8±10.4	Coronary angiogram, n (%)	30 (11.3)
Gender-male, (%)	213 (80.4)	PCI, n (%)	235(88.7)
Current smoker, n (%)	54 (20.4)	Angioseal, n (%)	60 (22.6)
Diabetes mellitus, n (%)	63 (23.8)	Perclose, n (%)	205 (77.4)
Hypertension, n (%)	130 (49.1)	Sheath size	
Hyperlipidemia, n (%)	147 (55.5)	6F, n (%)	237 (89.4)
Peripheral vascular disease, n (%)	8 (3.0)	7F, n (%)	2 (0.8)
		8F, n (%)	26 (9.8)
Renal failure, n (%)	7 (2.6)	Mean ACT, seconds	346±61
Left ventricular function (%)	64.9±14.3	Mean procedural time for archieving hemostasis, seconds	147±121
Body mass index, kg/m2	24.9±3.1		
		Right side : left side	256 : 7

The mean follow up time was 3320±628 days. Out of 233 patients with long term follow up, there was 1 case (0.4%) of Rutherford grade 2 claudication with a 79-year-old lady with end stage renal failure complained of bilateral calf claudication associated with rest pain. There was another case (0.4%) of bilateral Rutherford grade 1 claudication in a 78-year-old gentleman with bilateral lower limb peripheral vascular disease (PVD) and duplex ultrasound confirmed right superficial femoral artery (SFA) occlusion and left common femoral artery (CFA) 70% stenosis. The remaining patients were free of symptomatic PVD (Table 2).

Table 2 also showed the ABI and duplex ultrasound results on long term follow up. The ABI of accessed and non-accessed sites were 1.06±0.13 and 1.08±0.11

For peer review only - http://bmjopen.bmj.com/site/about/guidelines.xhtml

respectively. Duplex ultrasound measured peak systolic velocity of common femoral artery (CFA) in accessed and non-accessed sites were 87.43±22.34 cm/s and 87.69±22.06 cm/s respectively and of superficial femoral artery (SFA) were 81.40±20.85 cm/s and 81.31±17.80 cm/s respectively. There was no statistically significant difference in above parameters between accessed and non-accessed sites. Nevertheless, the mean end-diastolic diameter (EDD) of CFA and SFA in accessed sites was significantly larger than that in non-accessed sites (CFA: 8.12±1.27mm vs 7.80±1.16mm, *p*<0.01; SFA: 6.58±0.96mm vs 6.38±0.95mm, *p*<0.01).

Table 2 Follow up results

**Clinical Outcomes**			
**Rutherford's grade of claudication**			
**Grade 0, n (%)**	231 (99.2)		
**Grade 1, n (%)**	1 (0.4)		
**Grade 2, n (%)**	1 (0.4)		
	Access site	Control site	*p*
**Ankle Brachial Index (ABI)**	1.06±0.13	1.08±0.11	0.17
**Duplex ultrasound**	Access site	Control site	*p*
**CFA peak systolic velocity, cm/s**	87.43±22.34	87.69±22.06	0.73
**SFA peak systolic velocity, cm/s**	81.40±20.85	81.31±17.80	0.19
**CFA end-diastolic diameter, cm**	8.12±1.27	7.80±1.16	<0.01
**SFA end-diastolic diameter, cm**	6.58±0.96	6.38±0.95	<0.01

CFA: common femoral artery

SFA: superficial femoral artery

For peer review only - http://bmjopen.bmj.com/site/about/guidelines.xhtml

Data was also analysed between angioseal and perclose subgroup and results were shown in table 3. Baseline clinical and procedural characteristics were similar between both groups while patients received perclose had non-significantly higher peak systolic velocity in both CFA and SFA. The mean EDD of CFA in accessed sites was significantly larger in patients received perclose device than those received angioseal (perclose: 8.25±1.28mm vs angioseal: 7.58±1.10mm, p =0.02) but it is not the case for mean EDD of SFA.

Table 3 Angioseal vs Perclose

	Angioseal (n=60)	Perclose (n=205)	*P*
**Baseline characteristics**			
**Mean age (years)**	62.4±10.8	61.7±10.3	0.67
**Gender-male, (%)**	50 (83.3)	162 (79.8)	0.71
**Diabetes mellitus, n (%)**	19 (31.7)	44 (21.7)	0.12
**Hypertension, n (%)**	27 (45.0)	101 (49.8)	0.56
**Body mass index, kg/m2**	24.6±3.4	24.9±3.1	0.53
**Coronary angiogram, n (%)**	5 (8.5)	25 (12.5)	0.49
**PCI, n (%)**	55 (91.5)	178 (87.5)	0.49
**Parameters on accessed side**			
**Ankle Brachial Index (ABI)**	1.08±0.09	1.06±0.14	0.98
**CFA peak systolic velocity, cm/s**	84.66±15.09	88.07±23.70	0.55
**SFA peak systolic velocity, cm/s**	80.95±21.10	81.50±20.89	0.79
**CFA end-diastolic diameter, cm**	7.58±1.10	8.25±1.28	0.02
**SFA end-diastolic diameter, cm**	6.37±1.09	6.63±0.93	0.25

CFA: common femoral artery; SFA: superficial femoral artery

PCI: percutaneous coronary intervention

For peer review only - http://bmjopen.bmj.com/site/about/guidelines.xhtml

Discussion

To our knowledge, this is the first study to evaluate the long term impact of femoral vascular closure device on incidence of lower limb PVD. In the study published earlier, we demonstrated the favorable in-hospital and 1 year outcome of usage of FCD in our patients while this study showed nil evidence of increase in risk of lower limb PVD after a mean follow up of almost 10 years. Most previous studies including meta-analysis [1-5] comparing FCD and manual compression emphasized on evaluating short term vascular complications as safety outcome.. Theoretically, use of collagen plug and suture mediated vascular closure on arteriotomy site can impinge on the common femoral artery causing inflammation, remodeling and flow disturbances which potentially accelerates atherosclerosis. The process can take years to result in clinically significant artery narrowing. Our study using the non-accessed side as control and it identified nil increased incidence of clinical PVD by symptom, ABI and duplex ultrasound. The two patients suffered from symptomatic PVD had their right femoral artery accessed previously and the occurrence of bilateral lower limb arterial disease was unlikely accounted by use of FCD. On the contrary, the EDD of CFA and SFA in accessed sites in our patients were larger than that in non-accessed sites. Since we did not have baseline ultrasound of bilateral femoral arteries before device deployment, we cannot ascertain whether

For peer review only - http://bmjopen.bmj.com/site/about/guidelines.xhtml

the discrepancy is due to nature difference or device-related injury and positive remodeling. It has been shown in a study that diameter of right CFA is larger than that of left CFA [10],with a mean difference of 0.26mm which is similar to the difference of 0.32mm between our patients' accessed (>99% right side) and non-accessed sites. Further studies are needed to verify whether femoral closure device will have impact on femoral artery positive remodeling.

In animal model [11], collagen plug produced more intense inflammatory reaction of the tissue surrounding the femoral arteries when compared to suture mediated device but this did not lead to higher incidence of clinical PVD and duplex ultrasound derived flow abnormalities in our patients received Angioseal. On the other hand, the diameter of the accessed CFA is larger in perclose than in angioseal. We cannot determine whether a more intense inflammation with Angioseal gives rise to a smaller vessel caliber in the long run or Perclose triggers a more pronounced positive remodeling. Nonetheless, this interesting finding highlights tissue remodeling may differ between different mechanisms of vascular healing.

Limitations

We did not have baseline duplex ultrasound in our patients and we used non-accessed side as control for which we assumed the baseline appearance of both

For peer review only - http://bmjopen.bmj.com/site/about/guidelines.xhtml

sides of femoral arteries were identical. In addition, this was a single center study and the sample size was relatively small which may be underpowered to detect rare vascular events. Furthermore, more than one third of patients did not consent for ABI and duplex ultrasound measurement, making the data less representative. Moreover, VCD used in current study were either Angioseal or Perclose from 6 to 8 French and hence results may not be able to generalize to other VCD or in setting of larger size femoral access as in structural heart disease intervention.

Conclusions

Use of femoral vascular closure devices was safe and did not increase the incidence of lower limb peripheral vascular disease on long term follow up.

For peer review only - http://bmjopen.bmj.com/site/about/guidelines.xhtml

Contributors:

SWLL initiated the concept of work, performed the analyses in conjunction with coauthors and wrote and revised the manuscript. CCT, KLW, SLK, SYY, YTW and SYC performed clinical and ultrasound assessment. All authors contributed to the revisions of the manuscript and the interpretation of the findings.

Funding statement:

This research received no specific grant from any funding agency in the public, commercial or not-for-profit sectors

Competing interests:

None

Data Sharing Statement:

No additional data are available

For peer review only

For peer review only - http://bmjopen.bmj.com/site/about/guidelines.xhtml

References

1 Biancari F, D'Andrea V, Di Marco C, et al. Meta-analysis of randomized trials on the efficacy of vascular closure devices after diagnostic angiography and angioplasty. American heart journal. 2010;159(4):518-31. Epub 2010/04/07.

2 Chevalier B, Lancelin B, Koning R, et al. Effect of a closure device on complication rates in high-local-risk patients: results of a randomized multicenter trial. Catheterization and cardiovascular interventions : official journal of the Society for Cardiac Angiography & Interventions. 2003;58(3):285-91. Epub 2003/02/21.

3 Gerckens U, Cattelaens N, Lampe EG, et al. Management of arterial puncture site after catheterization procedures: evaluating a suture-mediated closure device. The American journal of cardiology. 1999;83(12):1658-63. Epub 1999/07/07.

4 Nikolsky E, Mehran R, Halkin A, et al. Vascular complications associated with arteriotomy closure devices in patients undergoing percutaneous coronary procedures: a meta-analysis. Journal of the American College of Cardiology. 2004;44(6):1200-9. Epub 2004/09/15.

5 Vaitkus PT. A meta-analysis of percutaneous vascular closure devices after diagnostic catheterization and percutaneous coronary intervention. The Journal of invasive cardiology. 2004;16(5):243-6. Epub 2004/05/21.

6 Kim YJ, Yoon HK, Ko GY, et al. Percutaneous transluminal angioplasty of suture-mediated closure device-related femoral artery stenosis or occlusive disease. The British journal of radiology. 2009;82(978):486-90. Epub 2008/12/23.

7 Lima S, Raposo L, Teles RC, et al. Femoral stenosis due to a collagen closure device treated percutaneously by peripheral stenting. Revista portuguesa de cardiologia : orgao oficial da Sociedade Portuguesa de Cardiologia = Portuguese journal of cardiology : an official journal of the Portuguese Society of Cardiology. 2009;28(7-8):877-80. Epub 2009/11/10.

8 Lee SW, Ho HH, Kong SL, et al. Long term clinical outcomes after deployment of femoral vascular closure devices in coronary angiography and percutaneous coronary intervention. Catheterization and cardiovascular interventions : official journal of the Society for Cardiac Angiography & Interventions. 2010;75(3):345-8. Epub 2009/11/26.

9 Rutherford RB, Baker JD, Ernst C, et al. Recommended standards for reports dealing with lower extremity ischemia: revised version. Journal of vascular surgery. 1997;26(3):517-38. Epub 1997/10/06.

10 Eisen LA, Minami T, Sekiguchi H, et al. Ultrasound demonstration of asymmetry between the left and right femoral and radial arteries. Chest. 2006;130(4):201s-s.

11 Gargiulo NJ, 3rd, Veith FJ, Ohki T, et al. Histologic and duplex comparison of the perclose and angio-seal percutaneous closure devices. Vascular. 2007;15(1):24-9.

For peer review only - http://bmjopen.bmj.com/site/about/guidelines.xhtml

Epub 2007/03/27.

For peer review only

For peer review only - http://bmjopen.bmj.com/site/about/guidelines.xhtml

Long Term Clinical Outcomes After Deployment of Femoral Vascular Closure Devices in Coronary Angiography and Percutaneous Coronary Intervention – A ~~Follow Up Cohort Study~~ single center registry follow up

Stephen Wai-Luen Lee, Chor-Cheung Tam, Ka-Lam Wong, Shun-Ling Kong, See-Yue Yung, Yiu-Tung Wong, Suk-Yee Chiu, Cheung-Chi Lam, Ki-Wan Chan, Hon-Wah Chan
Queen Mary Hospital, University of Hong Kong

Stephen Wai-Luen Lee
Division of Cardiology, Queen Mary Hospital, University of Hong Kong

Chor-Cheung Tam
Division of Cardiology, Queen Mary Hospital, University of Hong Kong

Ka-Lam Wong
Division of Cardiology, Queen Mary Hospital, University of Hong Kong

Shun-Ling Kong
Division of Cardiology, Queen Mary Hospital, University of Hong Kong

See-Yue Yung
Division of Cardiology, Queen Mary Hospital, University of Hong Kong

Yiu-Tung Wong
Division of Cardiology, Queen Mary Hospital, University of Hong Kong

Suk-Yee Chiu
Division of Cardiology, Queen Mary Hospital, University of Hong Kong

Cheung-Chi Lam
Division of Cardiology, Queen Mary Hospital, University of Hong Kong

Ki-Wan Chan
Division of Cardiology, Queen Mary Hospital, University of Hong Kong

Hon-Wah Chan

For peer review only
For peer review only - http://bmjopen.bmj.com/site/about/guidelines.xhtml

Division of Cardiology, Queen Mary Hospital, University of Hong Kong

Correspondence:
Stephen Wai-Luen Lee
1/F, A block, Queen Mary Hospital, 102, Pok Fu Lam Road, Hong Kong,
E-mail: leewls@ha.org.hk
Fax: 852-22555411

Keywords
Coronary intervention, coronary artery disease, adult cardiology, vascular closure device

Word count
1~~703~~851

For peer review only - http://bmjopen.bmj.com/site/about/guidelines.xhtml

ABSTRACT:

Objectives: There are few data evaluating the long term effect of femoral vascular closure devices (FCD) on patients' clinical outcome. We aim to evaluate the incidence of peripheral vascular disease (PVD) in patients received FCD following its deployment in coronary angiography and percutaneous coronary intervention (PCI) procedures.

Design and setting: Observational study of a Ssingle center, prospective cohort study registry

Participants: From June 2000 to September 2004, 265 patients who received FCD after coronary angiography and PCIs were enrolled into study.

Outcome measures: Clinical follow up (using Rutherford's categories of claudication), ankle brachial index (ABI) and Duplex ultrasound of femoral arteries (using non-accessed side as control) were performed to evaluate the presence of PVD.

Results: The mean follow up interval was 3320±628 days. 1 patient (0.4%) suffered from Grade 2 claudication and another (0.4%) suffered from Grade 1 claudication.

For peer review only

For peer review only - http://bmjopen.bmj.com/site/about/guidelines.xhtml

The mean ABI of accessed side and non-accessed side were 1.06±0.13 and 1.08±0.11 respectively, (p = 0.17). For duplex ultrasound, mean common femoral artery peak systolic velocity of accessed side and non-accessed side were 87.4±22.3 cm/s and 87.7±22.1 cm/s respectively, (p = 0.73); mean superficial femoral artery peak systolic velocity of accessed side and non-accessed side were 81.4±20.1 cm/s and 81.31±17.8 cm/s respectively, (p = 0.19).

Conclusion: The use of femoral vascular closure device after coronary angiogram and PCI is safe and does not increase the long term risk of peripheral vascular disease.

ARTICLE SUMMARY

For peer review only - http://bmjopen.bmj.com/site/about/guidelines.xhtml

Strengths and limitations of current study

- This study showed no increase in incidence of clinically significant peripheral vascular disease after use of femoral vascular closure device in long term follow up (median follow up time approaching 10 years).
- The use of duplex ultrasound showed no evidence of adverse effect of femoral vascular closure device on accessed femoral artery
- The lack of baseline ultrasound before vascular closure device precluded precise evaluation of effect of vascular remodeling of femoral artery after collagen plug and suture deployment.
- Current study results may not be able to generalize to other vascular closure device or in setting of large size femoral access as in structural heart disease intervention.

For peer review only

Introduction

Despite the increasing trend of utilizing radial access for coronary angiogram and percutaneous coronary intervention (PCI), femoral artery woundaccess hemostasis is still an integral part in the field of interventional cardiology. Femoral vascular closure devices (FCD) are designed specifically to facilitate arteriotomy closure which has been shown to allow shorter hemostasis time and early ambulation [1-3]. However, there is no data to demonstrate the superiority of the safetyof FCD in reducing vascular complications of FCD when compared with manual compression [1, 4, 5] and long term effect of FCD on peripheral vasculature has not been extensively investigated., apart from scanty case report describing the occurrence of delayed claudication post FCD use [6, 7]. Particularly, the impingement of femoral artery by FCD with subsequent inflammation and remodeling can take place for years which potentially cause vascular stenosis. Our group has previously published data [8] on the in-hospital and 1 year clinical outcomes in patients received FCD after coronary angiogram and PCIs before and we performed this follow up study to evaluate the long term clinical data incidence of lower limb peripheral vascular disease (PVD) in the same group of patients in long run.

Methods:

For peer review only - http://bmjopen.bmj.com/site/about/guidelines.xhtml

The design of the study has been described in elsewhere [8]~~-~~. In brief, ~~F~~from June 2000 to September 2004, 265 patients who successfully received FCDs after coronary angiogram and PCIs were enrolled for study. Femoral arteriogram were routinely performed before consideration of arterial closure and two types of FCD namely Angioseal (St. Jude Medical, Minnesota, MN) and Perclose (Abbott, Abbott Park, IL) closure devices were used. Angioseal is a collagen-mediated device which utilizes a bioabsorbable collagen sponge to seal the arteriotomy site while Perclose achieves hemostasis by delivering a knot to suture the arteriotomy.

For the long term registry follow up, all patients were seen in our clinic every four to six months and ~~The~~ occurrence of any vascular complications was recorded. ~~in-hospital and 1 year outcome was evaluated by clinical assessment and duplex ultrasound~~. ~~For the current study, patients were continued to be followed up in our clinic while c~~Clinical symptom and sign of lower limb ischemia were assessed by Rutherford's ~~categories~~grade of claudication [9]~~. Furthermore,~~ and ankle brachial index (ABI) was measured by using sphygmomanaometer and vascular doppler probe (Hadeco minidop ES-100VX). Patients were instructed to lie comfortably on bed and blood pressure cuff was applied to patients' arms and doppler probe was positioned at the brachial artery to obtain the systolic brachial pressure while the cuff was then placed at calfs with doppler probe positioned at dorsalis pedis or posterior tibial

For peer review only

For peer review only - http://bmjopen.bmj.com/site/about/guidelines.xhtml

artery (whichever is higher) to obtain the ankle systolic pressure. In addition, duplex ultrasound using a high frequency linear array transducer was performed to assess the peak systolic velocity at common femoral artery and superficial femoral artery. Non-accessed side was used as control to compare values of ABI and peak systolic velocity to the side where FCDs were deployed. The study was approved by the local ethics committee.

Continuous variables were expressed as the mean and standard deviation. Dichotomous variables were expressed as counts and percentage. Statistical comparisons were performed using Student's t test or Wilcoxon signed rank test for continuous variables as appropriate while Fisher's exact test was used for categorical variables. A P value < 0.05 was considered statistically significant. All statistical analyses were performed using SPSS for Windows (version 19.0, SPSS Inc.-------USA).

Results:

265 patients entered into study, the baseline clinical and procedural characteristics were shown in table 1. The mean age of patients was 61.8±10.4 years old with 80% were male. Vascular closure devices were used in 235 PCI (88.7%) and 30 (11.3%) coronary angiographic procedures. 60 procedures (22.6%) used Angioseal whereas

For peer review only - http://bmjopen.bmj.com/site/about/guidelines.xhtml

Perclose closure device was deployed in 205 procedures (77.4%). Most of the vascular closure devices used were 6F in size. Patients were continued to be followed up and for the current study, 233 patients were recruited for analysis while ABI and duplex ultrasound studies were performed in 145 patients (Figure 1).

Table 1 Baseline characteristics

Clinical characteristics		Procedural variables	
**Mean age (years)**	61.8±10.4	**Coronary angiogram, n (%)**	30 (11.3)
**Gender-male, (%)**	213 (80.4)	**PCI, n (%)**	235(88.7)
**Current smoker, n (%)**	54 (20.4)	**Angioseal, n (%)**	60 (22.6)
**Diabetes mellitus, n (%)**	63 (23.8)	**Perclose, n (%)**	205 (77.4)
**Hypertension, n (%)**	130 (49.1)	**Sheath size**	
**Hyperlipidemia, n (%)**	147 (55.5)	**6F, n (%)**	237 (89.4)
**Peripheral vascular disease, n (%)**	8 (3.0)	**7F, n (%)**	2 (0.8)
		**8F, n (%)**	26 (9.8)
**Renal failure, n (%)**	7 (2.6)	**Mean ACT, seconds**	346±61
**Left ventricular function (%)**	64.9±14.3	**Mean procedural time for archieving hemostasis, seconds**	147±121
**Body mass index, kg/m2**	24.9±3.1	**Right side : left side**	256 : 7

The mean follow up time was 3320±628 days. Out of 233 patients with long term follow up, there was 1 case (0.4%) of Rutherford grade 2 claudication with a 79-year-old lady with end stage renal failure complained of bilateral calf claudication associated with rest pain. There was another case (0.4%) of bilateral Rutherford grade 1 claudication in a 78-year-old gentleman with bilateral lower limb peripheral

For peer review only - http://bmjopen.bmj.com/site/about/guidelines.xhtml

vascular disease (PVD) and duplex ultrasound confirmed right superficial femoral artery (SFA) occlusion and left common femoral artery (CFA) 70% stenosis. The remaining patients were free of symptomatic PVD (Table 2).

Table 2 also showed the ABI and duplex ultrasound results on long term follow up. The ABI of accessed and non-accessed sites were 1.06±0.13 and 1.08±0.11 respectively. Duplex ultrasound measured peak systolic velocity of common femoral artery (CFA) in accessed and non-accessed sites were 87.43±22.34 cm/s and 87.69±22.06 cm/s respectively and of superficial femoral artery (SFA) were 81.40±20.85 cm/s and 81.31±17.80 cm/s respectively. There was no statistically significant difference in above parameters between accessed and non-accessed sites. Nevertheless, the mean end-diastolic diameter (EDD) of CFA and SFA in accessed sites was significantly larger than that in non-accessed sites (CFA: 8.12±1.27mm vs 7.80±1.16mm, $p<0.01$; SFA: 6.58±0.96mm vs 6.38±0.95mm, $p<0.01$).

For peer review only - http://bmjopen.bmj.com/site/about/guidelines.xhtml

Table 2 Follow up results

Clinical Outcomes			
**Rutherford's grade of claudication**			
**Grade 0, n (%)**	231 (99.2)		
**Grade 1, n (%)**	1 (0.4)		
**Grade 2, n (%)**	1 (0.4)		
	Access site	Control site	*p*
**Ankle Brachial Index (ABI)**	1.06±0.13	1.08±0.11	0.17
**Duplex ultrasound**	Access site	Control site	*p*
**CFA peak systolic velocity, cm/s**	87.43±22.34	87.69±22.06	0.73
**SFA peak systolic velocity, cm/s**	81.40±20.85	81.31±17.80	0.19
**CFA end-diastolic diameter, cm**	8.12±1.27	7.80±1.16	<0.01
**SFA end-diastolic diameter, cm**	6.58±0.96	6.38±0.95	<0.01

CFA: common femoral artery

SFA: superficial femoral artery

Data was also analysed between angioseal and perclose subgroup and results were shown in table 3. Baseline clinical and procedural characteristics were similar between both groups while patients received perclose had non-significantly higher peak systolic velocity in both CFA and SFA. The mean EDD of CFA in accessed sites was significantly larger in patients received perclose device than those received angioseal (perclose: 8.25±1.28mm vs angioseal: 7.58±1.10mm, p =0.02) but it is not the case for mean EDD of SFA.

For peer review only - http://bmjopen.bmj.com/site/about/guidelines.xhtml

Table 3 Angioseal vs Perclose

	Angioseal (n=60)	Perclose (n=205)	*P*
**Baseline characteristics**			
**Mean age (years)**	62.4±10.8	61.7±10.3	0.67
**Gender-male, (%)**	50 (83.3)	162 (79.8)	0.71
**Diabetes mellitus, n (%)**	19 (31.7)	44 (21.7)	0.12
**Hypertension, n (%)**	27 (45.0)	101 (49.8)	0.56
**Body mass index, kg/m2**	24.6±3.4	24.9±3.1	0.53
**Coronary angiogram, n (%)**	5 (8.5)	25 (12.5)	0.49
**PCI, n (%)**	55 (91.5)	178 (87.5)	0.49
**Parameters on accessed side**			
**Ankle Brachial Index (ABI)**	1.08±0.09	1.06±0.14	0.98
**CFA peak systolic velocity, cm/s**	84.66±15.09	88.07±23.70	0.55
**SFA peak systolic velocity, cm/s**	80.95±21.10	81.50±20.89	0.79
**CFA end-diastolic diameter, cm**	7.58±1.10	8.25±1.28	0.02
**SFA end-diastolic diameter, cm**	6.37±1.09	6.63±0.93	0.25

CFA: common femoral artery; SFA: superficial femoral artery

PCI: percutaneous coronary intervention

Discussion

To our knowledge, this is the first study to evaluate the long term ~~outcome~~ impact of femoral vascular closure device on incidence of lower limb PVD. In the study published earlier, we demonstrated the favorable in-hospital and 1 year outcome of usage of FCD in our patients while this study showed nil evidence of increase in risk of lower limb ~~peripheral vascular disease~~PVD after a mean follow up of almost 10 years. Most previous studies including meta-analysis [1-5] comparing FCD and manual compression ~~did not confirm a significant advantage of FCD in reducing~~

For peer review only - http://bmjopen.bmj.com/site/about/guidelines.xhtml

emphasized on evaluating short term vascular complications as safety outcome.apart from the shortened hemostasis time and faster ambulation of FCD over manual compression. Theoretically, use of collagen plug and suture mediated vascular closure after femoral arterial access on arteriotomy site can impinge on the common femoral artery causing inflammation, remodeling and potentially lead to flow disturbances which potentially accelerates atherosclerosis. The process can take years to result in clinically significant artery narrowing. Our study using the non-accessed side as control and it identified nil increased incidence of clinical PVD by symptom, ABI and duplex ultrasound. The two patients suffered from symptomatic PVD had their right femoral artery accessed previously and the occurrence of bilateral lower limb arterial disease was unlikely accounted by use of FCD. On the contrary, the EDD of CFA and SFA in accessed sites in our patients were larger than that in non-accessed sites. Since we did not have baseline ultrasound of bilateral femoral arteries before device deployment, we cannot ascertain whether the discrepancy is due to nature difference or device-related injury and positive remodeling. It has been shown in a study that diameter of right CFA is larger than that of left CFA [10],with a mean difference of 0.26mm which is similar to the difference of 0.32mm between our patients' accessed (>99% right side) and non-accessed sites. Further studies are needed to verify whether femoral closure

For peer review only - http://bmjopen.bmj.com/site/about/guidelines.xhtml

device will have impact on femoral artery positive remodeling.

In animal model [11], collagen plug produced more intense inflammatory reaction of the tissue surrounding the femoral arteries when compared to suture mediated device but this did not lead to higher incidence of clinical PVD and duplex ultrasound derived flow abnormalities in our patients received Angioseal. On the other hand, the diameter of the accessed CFA is larger in perclose than in angioseal. We cannot determine whether a more intense inflammation with Angioseal gives rise to a smaller vessel caliber in the long run or Perclose triggers a more pronounced positive remodeling. Nonetheless, this interesting finding highlights tissue remodeling may differ between different mechanisms of vascular healing.

Limitations

We did not have baseline duplex ultrasound in our patients and we used non-accessed side as control for which we assumed the baseline appearance of both sides of femoral arteries were identical. In addition, this was a single center study and the sample size was relatively small which may be underpowered to detect rare vascular events. Furthermore, more than one third of patients did not consent for ABI and duplex ultrasound measurement, making the data less representative. Moreover, VCD used in current study were either Angioseal or Perclose from 6 to 8

For peer review only - http://bmjopen.bmj.com/site/about/guidelines.xhtml

French and hence results may not be able to generalize to other VCD or in setting of larger size femoral access as in structural heart disease intervention.

Conclusions

Use of femoral vascular closure devices was safe and did not increase the incidence of lower limb peripheral vascular disease on long term follow up.

Contributors:

SWLL initiated the concept of work, performed the analyses in conjunction with coauthors and wrote and revised the manuscript. CCT, KLW, SLK, SYY, YTW and SYC performed clinical and ultrasound assessment. All authors contributed to the revisions of the manuscript and the interpretation of the findings.

Funding statement:

This research received no specific grant from any funding agency in the public, commercial or not-for-profit sectors

Competing interests:

None

For peer review only - http://bmjopen.bmj.com/site/about/guidelines.xhtml

For peer review only

References

1 Biancari F, D'Andrea V, Di Marco C, et al. Meta-analysis of randomized trials on the efficacy of vascular closure devices after diagnostic angiography and angioplasty. American heart journal. 2010;159(4):518-31. Epub 2010/04/07.

2 Chevalier B, Lancelin B, Koning R, et al. Effect of a closure device on complication rates in high-local-risk patients: results of a randomized multicenter trial. Catheterization and cardiovascular interventions : official journal of the Society for Cardiac Angiography & Interventions. 2003;58(3):285-91. Epub 2003/02/21.

3 Gerckens U, Cattelaens N, Lampe EG, et al. Management of arterial puncture site after catheterization procedures: evaluating a suture-mediated closure device. The American journal of cardiology. 1999;83(12):1658-63. Epub 1999/07/07.

4 Nikolsky E, Mehran R, Halkin A, et al. Vascular complications associated with arteriotomy closure devices in patients undergoing percutaneous coronary procedures: a meta-analysis. Journal of the American College of Cardiology. 2004;44(6):1200-9. Epub 2004/09/15.

5 Vaitkus PT. A meta-analysis of percutaneous vascular closure devices after diagnostic catheterization and percutaneous coronary intervention. The Journal of invasive cardiology. 2004;16(5):243-6. Epub 2004/05/21.

6 Kim YJ, Yoon HK, Ko GY, et al. Percutaneous transluminal angioplasty of suture-mediated closure device-related femoral artery stenosis or occlusive disease. The British journal of radiology. 2009;82(978):486-90. Epub 2008/12/23.

7 Lima S, Raposo L, Teles RC, et al. Femoral stenosis due to a collagen closure device treated percutaneously by peripheral stenting. Revista portuguesa de cardiologia : orgao oficial da Sociedade Portuguesa de Cardiologia = Portuguese journal of cardiology : an official journal of the Portuguese Society of Cardiology. 2009;28(7-8):877-80. Epub 2009/11/10.

8 Lee SW, Ho HH, Kong SL, et al. Long term clinical outcomes after deployment of femoral vascular closure devices in coronary angiography and percutaneous coronary intervention. Catheterization and cardiovascular interventions : official journal of the

For peer review only - http://bmjopen.bmj.com/site/about/guidelines.xhtml

Society for Cardiac Angiography & Interventions. 2010;75(3):345-8. Epub 2009/11/26.

9 Rutherford RB, Baker JD, Ernst C, et al. Recommended standards for reports dealing with lower extremity ischemia: revised version. Journal of vascular surgery. 1997;26(3):517-38. Epub 1997/10/06.

10 Eisen LA, Minami T, Sekiguchi H, et al. Ultrasound demonstration of asymmetry between the left and right femoral and radial arteries. Chest. 2006;130(4):201s-s.

11 Gargiulo NJ, 3rd, Veith FJ, Ohki T, et al. Histologic and duplex comparison of the perclose and angio-seal percutaneous closure devices. Vascular. 2007;15(1):24-9. Epub 2007/03/27.

For peer review only

For peer review only - http://bmjopen.bmj.com/site/about/guidelines.xhtml

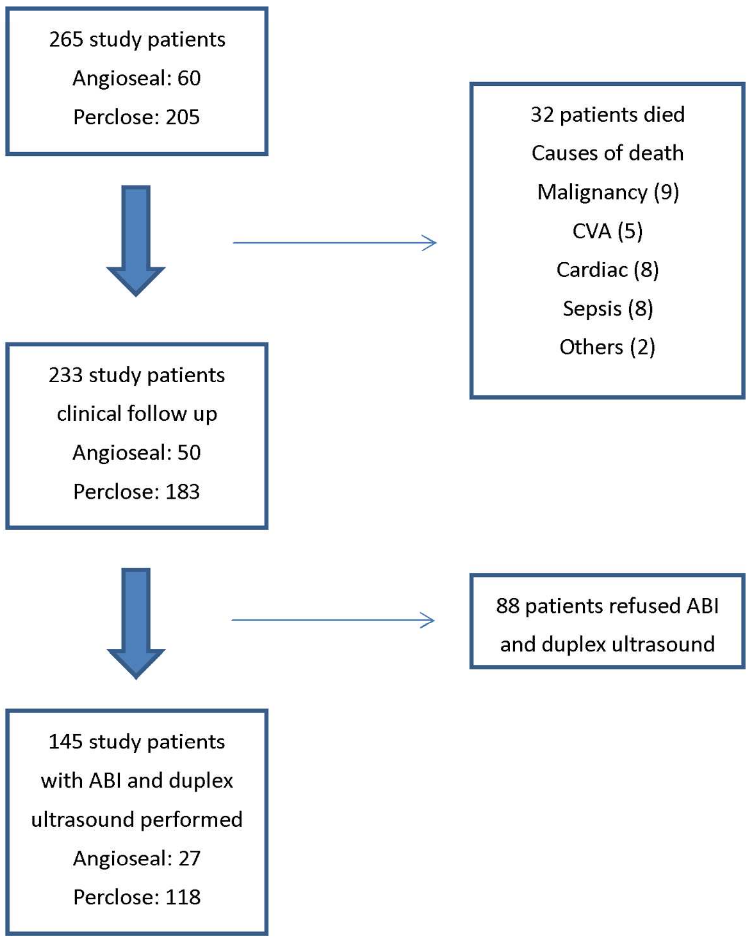

Figure 1 Study patients

142x196mm (300 x 300 DPI)

STROBE 2007 (v4) Statement—Checklist of items that should be included in reports of *cohort studies*

Section/Topic	Item #	Recommendation	Reported on page #
**Title and abstract**	1	(*a*) Indicate the study's design with a commonly used term in the title or the abstract	3, 6, 7
		(*b*) Provide in the abstract an informative and balanced summary of what was done and what was found	3, 4
**Introduction**			
Background/rationale	2	Explain the scientific background and rationale for the investigation being reported	6
Objectives	3	State specific objectives, including any prespecified hypotheses	6
**Methods**			
Study design	4	Present key elements of study design early in the paper	6, 7, reference 8
Setting	5	Describe the setting, locations, and relevant dates, including periods of recruitment, exposure, follow-up, and data collection	6, 7, reference 8
Participants	6	(*a*) Give the eligibility criteria, and the sources and methods of selection of participants. Describe methods of follow-up	6, 7, reference 8
		(*b*) For matched studies, give matching criteria and number of exposed and unexposed	
Variables	7	Clearly define all outcomes, exposures, predictors, potential confounders, and effect modifiers. Give diagnostic criteria, if applicable	6, 7
Data sources/ measurement	8*	For each variable of interest, give sources of data and details of methods of assessment (measurement). Describe comparability of assessment methods if there is more than one group	6, 7
Bias	9	Describe any efforts to address potential sources of bias	
Study size	10	Explain how the study size was arrived at	
Quantitative variables	11	Explain how quantitative variables were handled in the analyses. If applicable, describe which groupings were chosen and why	7
Statistical methods	12	(*a*) Describe all statistical methods, including those used to control for confounding	7
		(*b*) Describe any methods used to examine subgroups and interactions	
		(*c*) Explain how missing data were addressed	
		(*d*) If applicable, explain how loss to follow-up was addressed	
		(*e*) Describe any sensitivity analyses	
**Results**			
Participants	13*	(a) Report numbers of individuals at each stage of study—eg numbers potentially eligible, examined for eligibility, confirmed	Figure 1

For peer review only

		eligible, included in the study, completing follow-up, and analysed	
		(b) Give reasons for non-participation at each stage	Figure 1
		(c) Consider use of a flow diagram	Figure 1
Descriptive data	14*	(a) Give characteristics of study participants (eg demographic, clinical, social) and information on exposures and potential confounders	Table 1
		(b) Indicate number of participants with missing data for each variable of interest	
		(c) Summarise follow-up time (eg, average and total amount)	9
Outcome data	15*	Report numbers of outcome events or summary measures over time	9
Main results	16	(*a*) Give unadjusted estimates and, if applicable, confounder-adjusted estimates and their precision (eg, 95% confidence interval). Make clear which confounders were adjusted for and why they were included	
		(*b*) Report category boundaries when continuous variables were categorized	
		(*c*) If relevant, consider translating estimates of relative risk into absolute risk for a meaningful time period	
Other analyses	17	Report other analyses done—eg analyses of subgroups and interactions, and sensitivity analyses	
**Discussion**			
Key results	18	Summarise key results with reference to study objectives	12
**Limitations**			
Interpretation	20	Give a cautious overall interpretation of results considering objectives, limitations, multiplicity of analyses, results from similar studies, and other relevant evidence	12, 13, 14
Generalisability	21	Discuss the generalisability (external validity) of the study results	13, 14
**Other information**			
Funding	22	Give the source of funding and the role of the funders for the present study and, if applicable, for the original study on which the present article is based	14, 15

*Give information separately for cases and controls in case-control studies and, if applicable, for exposed and unexposed groups in cohort and cross-sectional studies.

Note: An Explanation and Elaboration article discusses each checklist item and gives methodological background and published examples of transparent reporting. The STROBE checklist is best used in conjunction with this article (freely available on the Web sites of PLoS Medicine at http://www.plosmedicine.org/, Annals of Internal Medicine at http://www.annals.org/, and Epidemiology at http://www.epidem.com/). Information on the STROBE Initiative is available at www.strobe-statement.org.

For peer review only
